# Words or numbers? Communicating risk of adverse effects in written consumer health information: a systematic review and meta-analysis

**DOI:** 10.1186/1472-6947-14-76

**Published:** 2014-08-26

**Authors:** Roland Brian Büchter, Dennis Fechtelpeter, Marco Knelangen, Martina Ehrlich, Andreas Waltering

**Affiliations:** 1 Department of Health Information, Institute for Quality and Efficiency in Health Care (IQWiG), Im Mediapark 8, 50670 Cologne, Germany

**Keywords:** Consumer health information, Health communication, Risk, Meta-analysis, Review

## Abstract

**Background:**

Various types of framing can influence risk perceptions, which may have an impact on treatment decisions and adherence. One way of framing is the use of verbal terms in communicating the probabilities of treatment effects. We systematically reviewed the comparative effects of words versus numbers in communicating the probability of adverse effects to consumers in written health information.

**Methods:**

Nine electronic databases were searched up to November 2012. Teams of two reviewers independently assessed studies. Inclusion criteria: randomised controlled trials; verbal versus numerical presentation; context: written consumer health information.

**Results:**

Ten trials were included. Participants perceived probabilities presented in verbal terms as higher than in numeric terms: commonly used verbal descriptors systematically led to an overestimation of the absolute risk of adverse effects (Range of means: 3% - 54%). Numbers also led to an overestimation of probabilities, but the overestimation was smaller (2% – 20%). The difference in means ranged from 3.8% to 45.9%, with all but one comparison showing significant results. Use of numbers increased satisfaction with the information (MD: 0.48 [CI: 0.32 to 0.63], p < 0.00001, I^2^ = 0%) and likelihood of medication use (MD for very common side effects: 1.45 [CI: 0.78 to 2.11], p = 0.0001, I^2^ = 68%; MD for common side effects: 0.90 [CI: 0.61 to 1.19], p < 0.00001, I^2^ = 1%; MD for rare side effects: 0.39 [0.02 to 0.76], p = 0.04, I^2^ = not applicable). Outcomes were measured on a 6-point Likert scale, suggesting small to moderate effects.

**Conclusions:**

Verbal descriptors including “common”, “uncommon” and “rare” lead to an overestimation of the probability of adverse effects compared to numerical information, if used as previously suggested by the European Commission. Numbers result in more accurate estimates and increase satisfaction and likelihood of medication use. Our review suggests that providers of consumer health information should quantify treatment effects numerically. Future research should focus on the impact of personal and contextual factors, use representative samples or be conducted in real life settings, measure behavioral outcomes and address whether benefit information can be described verbally.

## Background

Ideally, patient decisions for and against medical treatments are made in the presence of knowledge of the best available evidence for the benefits and harms of these treatments. Personal preferences and values can influence treatment decisions and may – legitimately – lead people to make choices which are not necessarily in line with the evidence. There are, however, some cognitive biases that may interfere with treatment. In particular, various types of data framing can influence risk perceptions [1].

Poorly framed information on the risk of adverse effects of drugs or other medical interventions may cause misinterpretation of the risks of harms. This may have an impact on treatment decisions and might also affect medication adherence. The 1995 contraceptive pill scare in the UK highlights the importance of helping doctors and patients understand risk information: media reports and “Dear Doctor” letters reported that the third-generation contraceptive pills increased the (relative) risk of blood clots by 100%, which caused many women to stop taking the pill and led to many unwanted pregnancies and abortions – although the absolute risk increase was as small as 0.014% [2].

One way of framing information is the use of words in communicating the probabilities of treatment effects. A prominent example for a nomenclature of words used to communicate frequencies of adverse effects is the one proposed by the European Commission in their 1998 guidelines on the readability of package leaflets and summary of product characteristics from the European Medical Association [3,4]. Table 1 shows the wording suggested in these guidelines.

**Table 1:** European commission nomenclature for communicating frequency of adverse effects of drugs

**Description**	**Frequency interval**
Very common	(≥1/10)
Common	(≥1/100 to <1/10)
Uncommon	(≥1/1000 to <1/100)
Rare	(≥1/10000 to <1/1000)
Very rare	(<1/10000)
Not known	cannot be estimated from the available data

Several studies have compared the use of verbal terms versus numbers for communicating the frequency of adverse drug effects. However, to our knowledge no systematic review on the comparative effects of verbal versus numerical presentations of the frequency of adverse effects has been conducted. Risk communication has become a vast field which is difficult to keep up with. Thus, current recommendations on risk communication are often based on expert consensus or a selective review of the literature. For example, both the International Patient Decision Aid Standards (IPDAS) and the FDA’s user’s guide on communicating risks and benefits currently do not cite many of the studies we identified in our preliminary searches. The aim of this systematic review is to improve the evidence base of risk communication strategies by gathering and synthesizing the results from studies that examined different terms, scenarios and probabilities.

## Methods

### Inclusion criteria

We included studies examining the effects of words versus numbers in communicating harms of treatments to consumers in written health information. Our inclusion criteria were: (1) study design: randomized controlled trials (RCTs); (2) outcomes: interpretation of probability, comprehension, recall, satisfaction, impact on decision, likelihood of treatment utilization, adherence and psychological outcomes (e.g. anxiety); (3) context: treatment effects were communicated through written health information only and (4) language: studies published in English or German.

### Data sources and search methods

We searched MEDLINE, Embase, PsycINFO, CINAHL, ERIC, DARE, the CDSR, CENTRAL and the Campbell Library. Searches were developed and conducted by an information specialist using a combination of MeSH-terms, free text and validated search filters for specific study designs, where available. See Additional file 1 for the search strategy used to identify relevant studies in MEDLINE. This was adapted as required to other databases. Searches were conducted up to the 9th of November 2012. Titles and abstract of search results were assessed for eligibility independently by three reviewers in pairs. Full texts of potentially relevant studies were retrieved and assessed for eligibility independently by two reviewers. Reference lists of articles eligible for inclusion were screened for further potentially relevant studies.

### Data extraction and risk of bias assessment

Data were extracted into standardized extraction sheets and double checked in pairs by three reviewers. These included data on study design, risk of bias items, population characteristics, study setting, study intervention and results for the relevant outcomes (means and standard deviations). In studies that only reported p-values, t-values or confidence intervals, we derived standard deviations from these statistics using the methods described in Chapter 7 of the Cochrane Handbook for Systematic Reviews [5].

Risk of bias was assessed for RCTs by random sequence generation, allocation concealment, completeness of follow-up and selective reporting bias. Judgements were made in accordance with the guidelines for the Cochrane risk of bias tool [6].

### Data synthesis and analysis

Data were entered into RevMan 5 and pooled. Mean differences (MD) and their corresponding 95% confidence intervals (CI) were calculated for outcomes that were measured on scales of considerable similarity. Otherwise standardised mean differences were calculated. Meta-analyses were conducted using random-effects models as the underlying rationale of random-effects models may be more appropriate when pooling heterogeneous data, while fixed and random-effects models produce the same result if data are homogenous. A downside of random-effects models is that more weight is given to small studies which may have a higher risk of bias (small study bias), but this was not an issue in our review. Heterogeneity was measured using Chi^2^-tests and the I^2^ statistic. If heterogeneity was detected, subgroup analyses were conducted to explore reasons for heterogeneity. Subgroups were planned a priori for age, gender, socioeconomic status, type of illness (mild or severe), size of absolute effect and severity of side effects. Where statistical heterogeneity remained, but there was strong contextual homogeneity, we opted in favour of pooling the data into meta-analyses, because of their additional informational value and the problems associated with narrative or pseudo-quantitative interpretation of results [7]. However, in these cases we did not pool results across subgroups.

Some studies had three comparison groups: two studies compared a verbal, percentage and natural frequency presentation; one study compared a verbal, numerical and combined verbal/numerical presentation [8]. In this case we used data from both comparisons in our analyses and divided the number of participants in the verbal group by two in order not to artificially inflate the statistical power of these studies in the meta-analyses. In two studies participants received two scenarios with different adverse effects. In cases where both scenarios were relevant to the same meta-analysis, we averaged the results across the two scenarios. The standard deviations for these comparisons were recalculated to account for statistical dependence assuming a correlation of 0.5 (sensitivity analyses with correlations of 0.1 and 0.9 produced similar results).

## Results

### Search results

**Figure 1:**
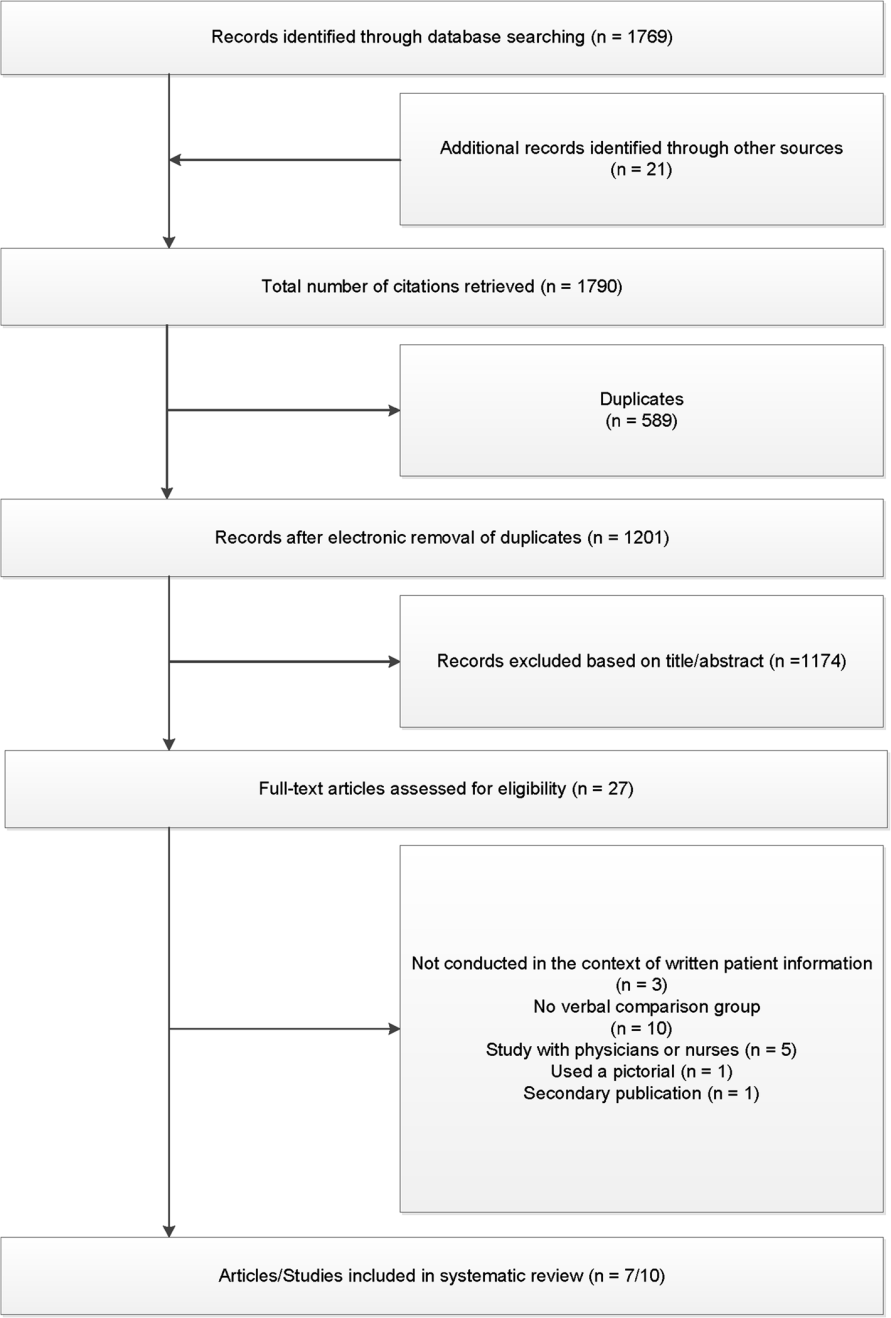
**Study selection process.** Flow-chart showing the study selection procedure according to PRISMA reporting guidelines.

Figure 1 shows a flow diagram depicting the study selection process in accordance with the PRISMA statement [9]. Our searches yielded 1201 potentially relevant articles. Seven articles including ten studies remained eligible for inclusion after applying the inclusion criteria [8,10-15].

### Description of studies

All studies were randomized controlled trials, many of which used a factorial design. Some studies were reported in more than one publication. All studies randomized participants to short information leaflets on drugs for a particular condition, which only differed in whether the information on the frequency of the adverse effects of the drug were presented verbally or numerically. One study examined a combination of a verbal and numerical description, as it is currently included in the 2009 European Commission Guideline on the readability of package leaflets [16]. The interventions and outcomes of the studies were very similar and mainly differed with respect to the conditions and drugs that were used in the scenarios as well as the frequency and the severity of the side effects. The studies included five outcomes of interest to our review: estimation of probabilities (in percentages), likelihood of occurrence, satisfaction, intention to take or continue to take the medicine and the impact of the information on the decision. The last four outcomes were all measured as one item on a 6-point Likert scale. All outcomes were measured shortly after distribution of the information leaflets, and none of the studies had a follow-up. In many cases the participants received information on more than one adverse effect, resulting in a higher number of comparisons than studies for the outcome estimation of probability.

In all but one study participants were recruited from the general population or via a cancer website and confronted with a hypothetical scenario. The studies were all conducted by two groups of authors from the UK, who were interested in evaluating the effects of the nomenclature used in drug package inserts in the European Union. Thus, the verbal descriptors that were studied in the trials were: very common, common, uncommon, rare and very rare. See Additional file 2 for detailed characteristics of the included studies with additional results from individual studies regarding effect modifiers.

### Risk of bias

Methods of sequence generation, allocation concealment and information on incomplete outcome data were frequently not reported. Thus, none of the studies was formally rated low on all risk of bias items (Table 2). Nevertheless we consider the overall risk of bias to be low. For one thing, there was a large overlap in the group of authors, suggesting that the methods used were likely to be appropriate despite not being fully reported in each study – especially considering that unconcealed allocation was explicitly acknowledged in one study. For another thing, the design of the studies was rather simple. Participants completed questionnaires immediately after reading the information in the presence of the researchers. Therefore, it can be assumed that missing data were not an issue even if this was not explicitly stated for each item in all publications.

**Table 2:** Risk of bias of included studies

**Study**	**Random sequence generation**	**Allocation concealment**	**Incomplete outcome data**	**Selective reporting**
Berry 2002 Study 1 [10]	Unclear	Unclear	Low	Low
Berry 2002 Study 2 [10]	Unclear	Unclear	Low	Low
Berry 2003 Study 1 [11]	Unclear	Unclear	Low	Low
Berry 2003 Study 2 [11]	Unclear	Unclear	Low	Low
Berry 2004 [12]	Unclear	Unclear	Low	Low
Berry 2006 [13]	Unclear	Unclear	Unclear	Low
Knapp 2004 [14]	Unclear	High	Unclear	Low
Knapp 2009a [8]	Low	Low	Unclear	Low
Knapp 2009b Study 1 [8]	Low	Low	Unclear	Low
Knapp 2009 Study 2 [15]	Low	Low	Unclear	Low

One study used an unconcealed allocation. However, the authors of the study argued that this was unlikely to bias the results, because it seems unlikely that the researcher could be able to anticipate the participants’ response to verbal or numerical information. Furthermore, excluding this study did not alter the results. There were no signs of selective reporting.

### Effects of interventions

#### ***Estimation of probabilities***

**Figure 2:**
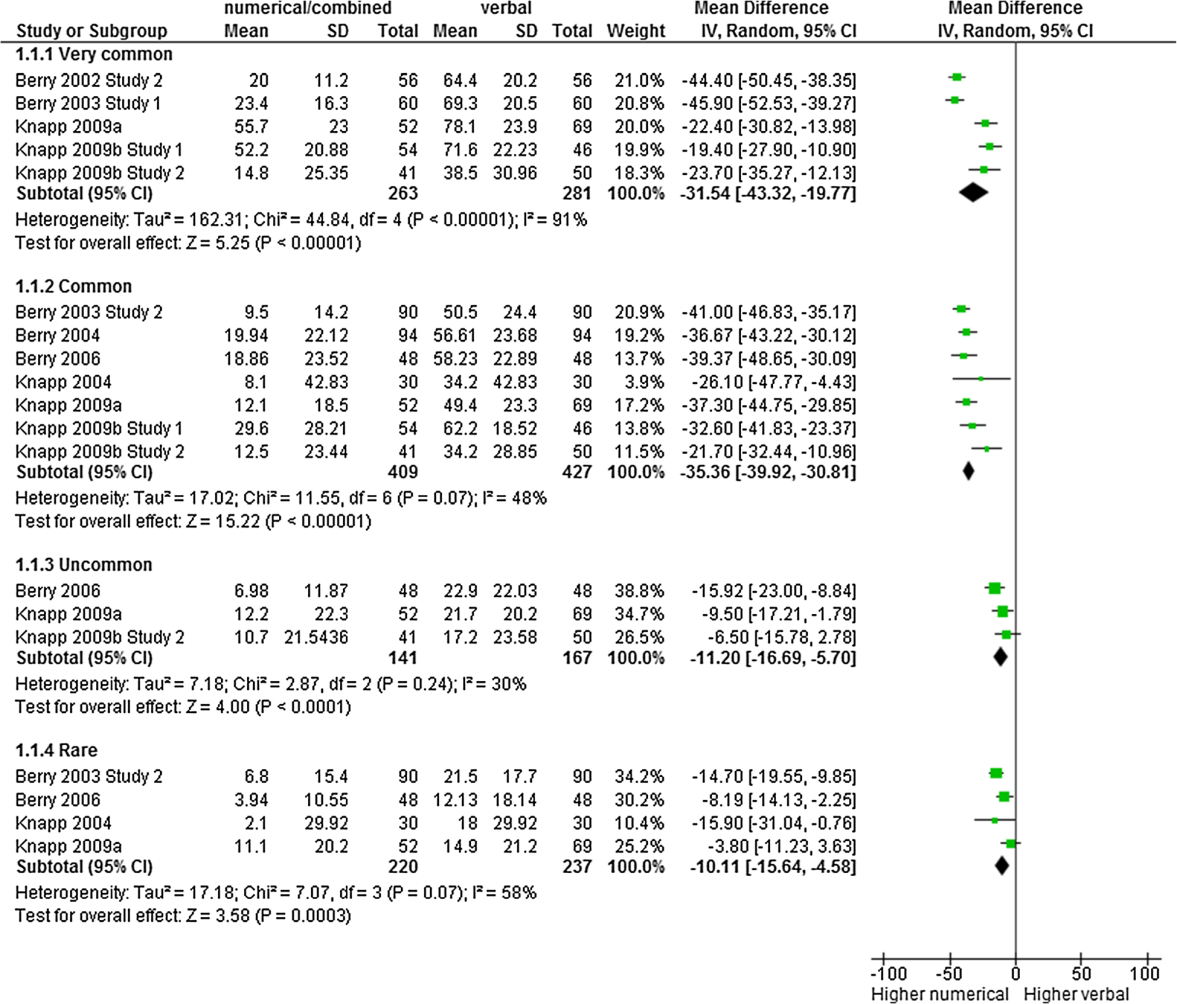
**Estimation of frequency.** Meta-analysis on estimation of frequency of adverse effects.

There were 19 comparisons from 10 studies with 2145 observations for the outcome estimation of probabilities. The verbal descriptors used for communicating frequencies of adverse effects systematically led to an overestimation of the probability of adverse effects compared to a numerical presentation (MD for very common side effects: -31.54 [CI: -43.32 to -19.77], p < 0.00001, I^2^ = 91%; MD for common side effects: -35.36 [CI: -39.92 to -30.81], p < 0.00001, I^2^ = 48%;%; MD for uncommon side effects: -11.20 [-16.69 to -5.70], p < 0.0001, I^2^ = 30%; MD for rare side effects: -10.11 [-15.64 to -4.58], p = 0.0003, I^2^ = 58%). The absolute magnitude of the overestimation was larger in the very common and common subgroup than in the uncommon and rare subgroup, as it would be expected (Figure 2). Subgroup analyses by frequency did not fully explain the heterogeneity. The differences in frequencies used in the studies are likely to contribute to this heterogeneity (see Additional file 3).

Interestingly, even participants who received a probability estimate of the frequency of the adverse effects often overestimated these values. Only between 9% and 50% of the participants in the numerical groups gave a correct probability for the adverse effects (see Additional file 3). However, this was not always reported. Furthermore, the variability in responses between participants was large, which is indicated by large standard deviations and wide ranges.

See Additional file 3 for a detailed table of the results of the comparisons from each study by verbal descriptor and type and frequency of adverse effect together with the results of the significance tests as they were reported in the primary studies.

### Likelihood of occurrence

**Figure 3:**
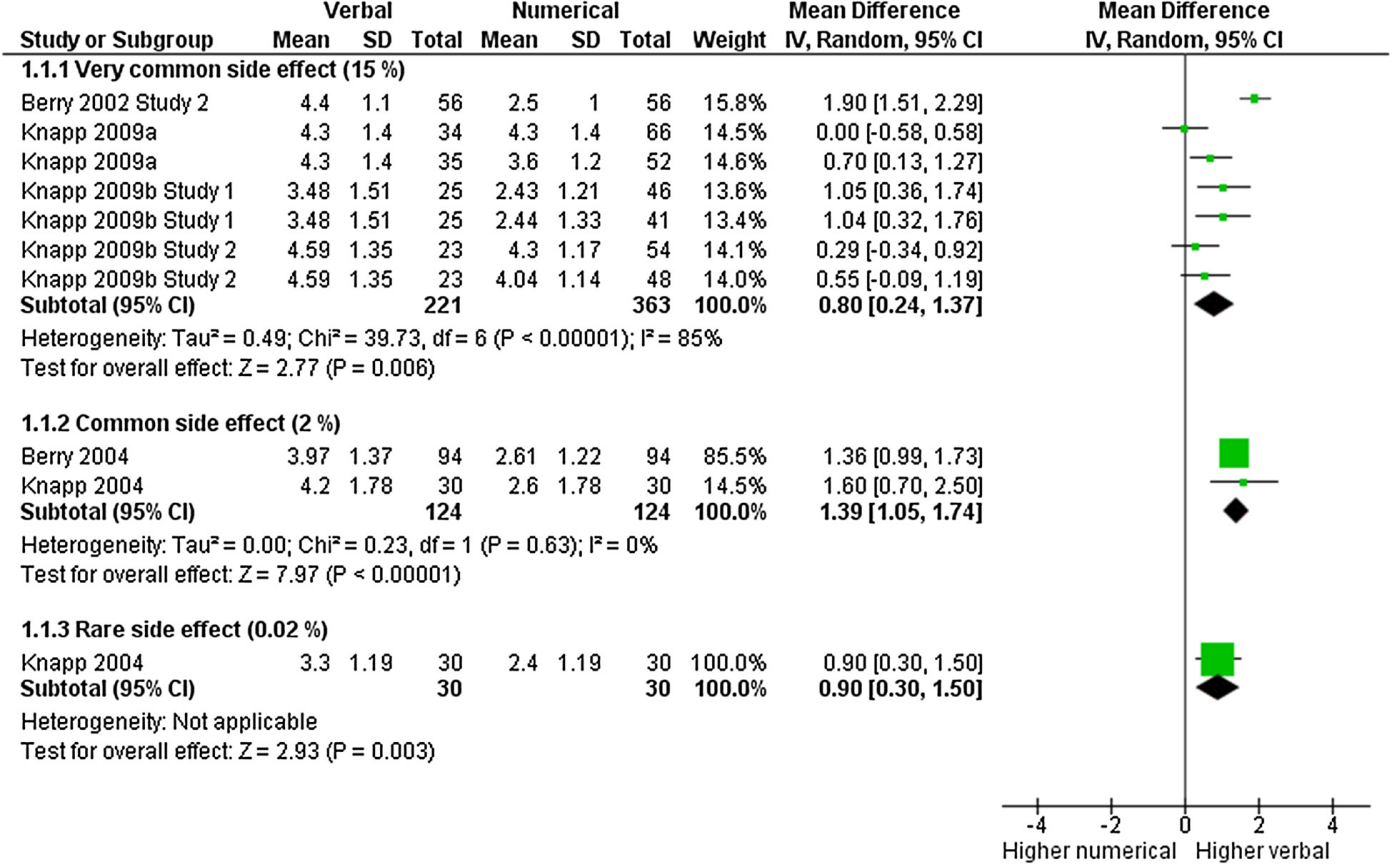
**Likelihood of occurrence.** Meta-analysis on perceived likelihood of occurrence.

Likelihood of occurrence as measured on a Likert scale was considered in 10 comparisons with 892 observations. Participants who received a verbal presentation of the frequency of adverse effects thought they were more likely to occur than those who received numerical information (MD for very common side effects: 0.80 [CI: 0.24 to 1.37], p = 0.006, I^2^ = 85%; MD for common side effects: 1.39 [CI: 1.05 to 1.74], p < 0.00001, I^2^ = 0%; MD for rare side effects: 0.90 [0.30 to 1.50], p = 0.003, I^2^ = not applicable). We conducted a subgroup analysis by frequency of adverse effect, but this did not fully explain the large heterogeneity for this outcome (Figure 3). However, the heterogeneity can mainly be attributed to one trial [10], which showed a large difference and included postgraduate or undergraduate students in contrast to the other studies, which included participants from the general public. Excluding this trial from the analysis reduces the heterogeneity in the respective subgroup from 85% to 39%.

One trial compared a numerical presentation with a combined format. Splitting this trial from the others we conducted a second, exploratory subgroup analysis on this outcome. This suggested that the verbal presentation may dilute the effects of a numerical presentation on this outcome (test for subgroup difference, p = 0.003, analysis not shown) [15].

### Satisfaction

**Figure 4:**
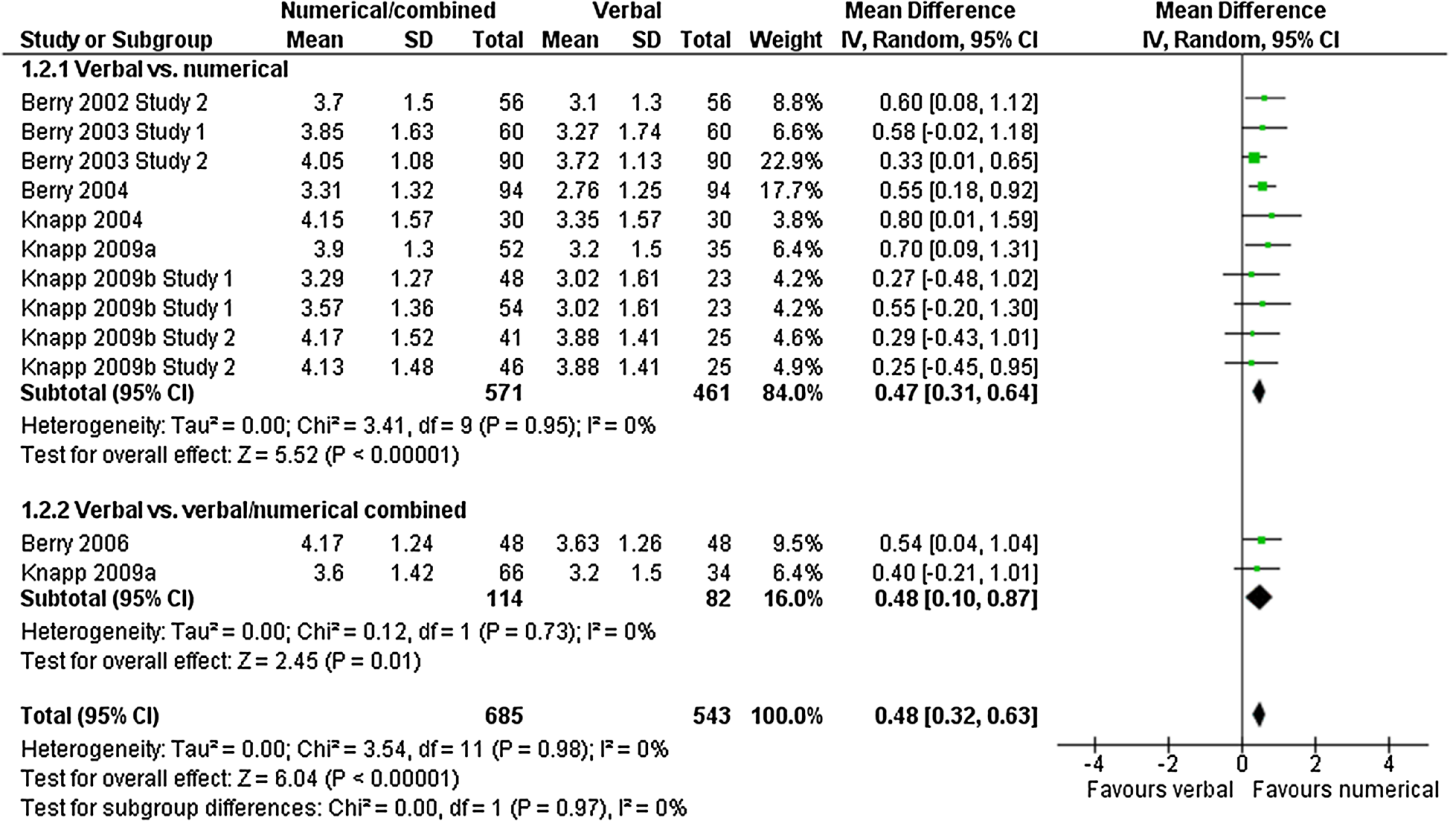
**Satisfaction with information.** Meta-analysis on satisfaction with information.

Satisfaction with the information was measured in 12 comparisons with 1228 observations. Satisfaction was consistently higher in groups that received a numerical description of the frequency of adverse effects compared to a verbal description (MD: 0.48 [CI: 0.32 to 0.63], p < 0.00001, I^2^ = 0%) (Figure 4).

### Likelihood of taking the medicine

**Figure 5:**
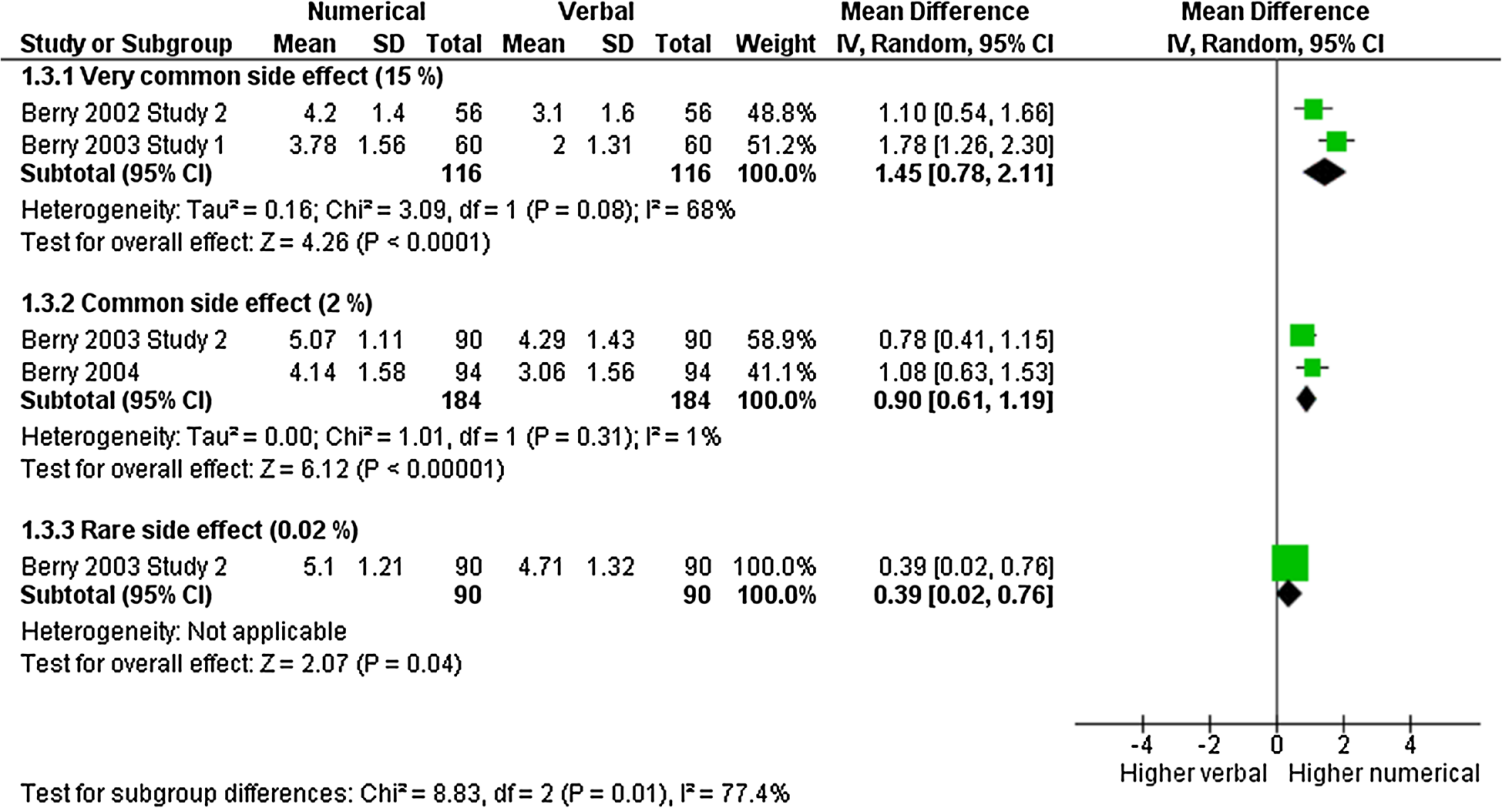
**Likelihood of (continuing) taking drug.** Meta-analysis on likelihood of (continuing) taking the drug.

Data for the outcome likelihood of taking or continuing to take the drug in the scenario was available from 5 comparisons with 780 observations. Participants who were presented with numbers, stated that they were or would be more likely to take or continue taking the drugs which were suggested to them (MD for very common side effects: 1.45 [CI: 0.78 to 2.11], p < 0.0001, I^2^ = 68%; MD for common side effects: 0.90 [CI: 0.61 to 1.19], p < 0.00001, I^2^ = 1%; MD for rare side effects: 0.39 [0.02 to 0.76], p = 0.04, I^2^ = not applicable) (Figure 5). There was a significant effect for subgroup differences according to the frequency of the adverse effect (p = 0.01). Based on the EU nomenclature, this suggests that the larger the frequency of the adverse effect, the less likely it is that participants will take the drug, if they are presented with a verbal format.

### Impact of information on decision

**Figure 6:**
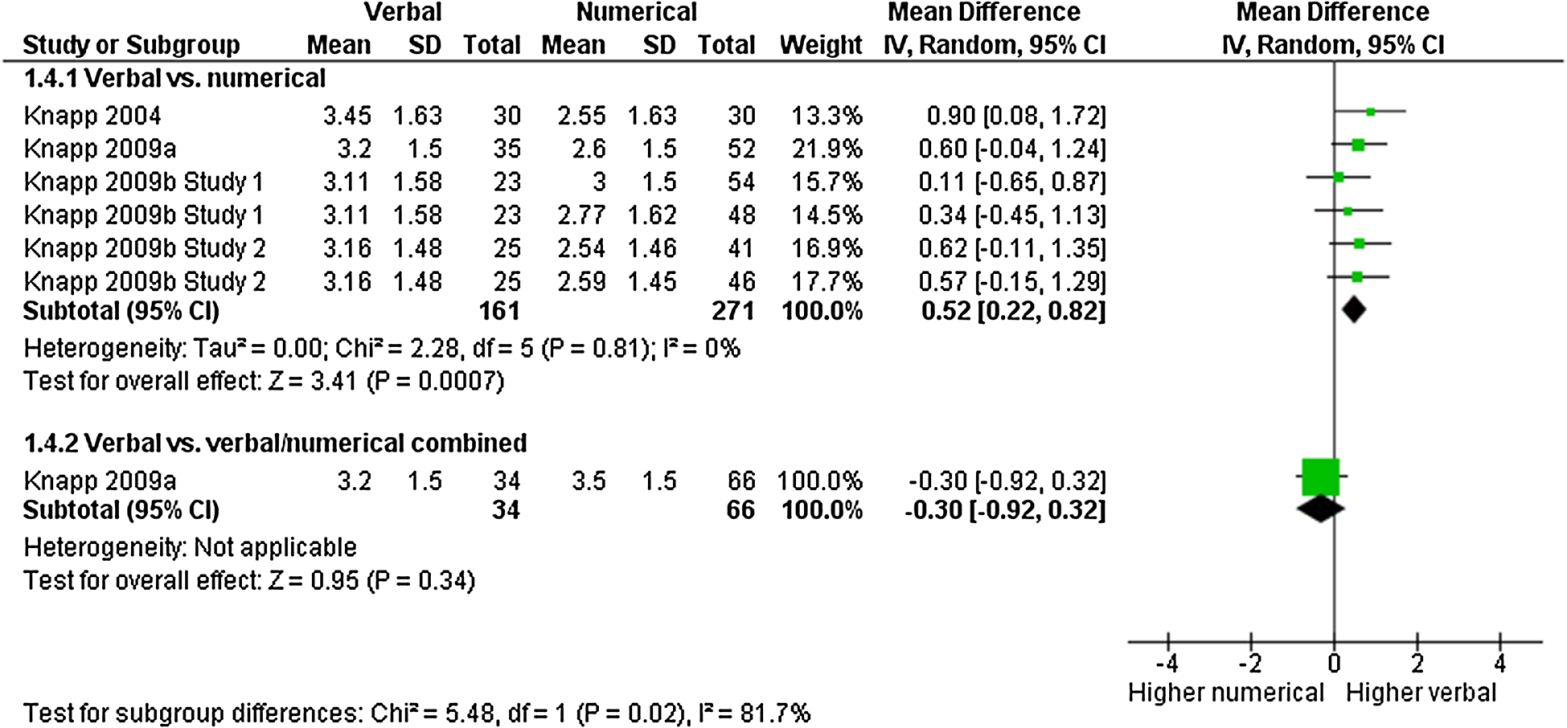
**Impact on decision.** Meta-analysis on impact of information on (hypothetical) decision.

The impact of the information on the decision to take or continue to take the medication was measured in 7 comparisons with 532 observations. Verbal presentations of adverse effects had a larger impact on the decision to take the drugs than numerical presentations (MD: 0.52 [CI: 0.22 to 0.82], p = 0.0007, I^2^ = 0%) (Figure 6). There was a significant subgroup effect for the difference between a numerical and a combination of numerical and verbal presentation, suggesting that the verbal presentation may dilute the effects of a numerical presentation on this outcome (test for subgroup differences p = 0.02). However, this subgroup analysis is restricted to one study and was conducted post hoc, so the results should be interpreted with caution.

## Discussion

This systematic review provides evidence that compared to numerical information verbal descriptors commonly used to communicate the frequencies of adverse effects in written health information including “common”, “uncommon” and “rare” lead to an overestimation of the probability of adverse effects, when they are used as previously suggested in the Guidelines of the European Commission.

It could be argued that other verbal terms are needed to describe frequencies. We are not aware of any studies comparing verbal terms other than those suggested in the 1998 European Commission’s guidelines though. Some studies have asked patients to assign probability values to a range of different verbal frequency terms [17]. According to these studies, other words do not appear to be better suited to describe frequencies than those previously suggested by the European Commission. For example, in one study in a general practice setting, the terms “almost never” and “rarely” were associated with the lowest frequencies [18]. The probabilities assigned to these terms were still very high with 9.9% and 7.5%, respectively. Furthermore, the standard deviations in these studies were large, which is in line with our results and suggests a large variance in the frequencies assigned to different terms. This indicates that risk expressions should be tested for understanding before being routinely used. Furthermore, it suggests that there may be no verbal labels that are suited to convey frequencies, particularly of rare adverse effects.

Even participants who received numerical information overestimated the risk of adverse effects. This is in line with other findings showing that people are generally poor at estimating risks [19]. Low numeracy in some of the patients may also explain this finding. In the UK, for example, one study suggested that one third of adults above the age of 50 had limited functional health literacy [20]. Another possible explanation for this finding is that patients may perceive their personal risk of experiencing adverse effects to be larger than average.

People seem to be more satisfied with numerical presentations and that they would be more likely to take the drugs or continue taking them. Participants also stated that they would be less affected in their decisions by numerical presentations. These outcomes were measured on a 6-point Likert scale. Converting difference into percentages on the scale suggests changes between 7% and 24%, which can be considered to be in the small to moderate, but important range. Most effects were also in the small to moderate range based on Cohen’s interpretation, when converting effects into standardised mean differences. Some of the effects may be considered relatively large, since there is a tendency for people to avoid extreme answers on scales where extreme values are labelled in absolute terms, as it was the case in the studies included in this article [21].

Subgroup analyses suggested that combined verbal and numerical formats may dilute the effects of the numerical presentation on two outcomes, namely likelihood of occurrence (as measure on a Likert scale) and impact on treatment decision. However, these results are based on a post-hoc analysis and comparison with the combined format was restricted to a single trial with 100 participants.

### Challenges for providers of patient information

Providers of patient information often have a broad audience and face the problem that people have different preferences regarding the need and use of risk estimates. The meaning that is ascribed to such information varies greatly. While some express a clear need for risk estimates, others are confused by numbers and prefer to make decisions based on other types of information [22]. Different preferences imply that using a combined verbal and numerical format may be the best compromise to suit various needs. This is also reflected in the current European Commission Guideline on readability from 2009 as well as the current EU template for patient leaflets [16,23]. Providing different information for different groups according to their preferences would be an option, but it may be difficult to direct patients to the information that best suits their needs.

Unfortunately, data on adverse effects are often poorly reported in trials and systematic reviews, which complicates the issue [24,25]. Furthermore, there might still be a role for verbal terms in written information, for example for people with difficulties in understanding numbers, or when large amounts of numbers make information too difficult to comprehend. It is difficult to draw a clear recommendation for providers of patient information as it is unlikely that there is a one-size-fits-all approach. This will depend on many other factors such as the context and the target group of the information.

### Limitations of the review

Our review is based on a comprehensive search and used rigorous methods for assessing and synthesising the included studies. However, it has some limitations. We restricted our search to English and German studies. It is reported in accordance with the PRISMA statement (Additional file 4). This may have introduced a language bias. We do not consider this to be a major weakness though, since it is questionable whether results can be generalized from one language to another due to semantic differences.

### Limitations of the included studies

Many studies were conducted with healthy volunteers and used fictional scenarios. There were some exceptions: one study included patients admitted to a cardiac rehabilitation centre and produced similar results. Three studies of users of a patient information website partially included women with experiences of breast cancer. While they also produce similar results, these trials had some limitations, too. Some of the women in these studies were already taking the medication which was used in the scenario, which questions the applicability to other populations. An important caveat of all studies was that they used convenience samples, which may lack representativeness. Lastly, all outcomes were measured as single items. This may be problematic for an outcome such as satisfaction, which represents a complex construct. However, information leaflets only differed in one sentence and the results for this outcome were very homogenous, adding strength to the findings.

## Conclusions

Our review suggests that – whenever possible – adverse treatment effects should be quantified numerically, because they lead to better estimates of risks. Verbal risk expressions should be tested for understanding before being routinely used.

Further studies should focus on the impact of personal and contextual factors, including the setting, disease, numeracy and educational level. Furthermore they should use representative samples or be conducted in real life settings and measure potentially more relevant outcomes such as actual behavior (including decisions and medication adherence for example) and whether decisions are in line with personal values. After all, risk communication is not an end in itself, but a means to the end of making better decisions. On a more critical note, it is questionable whether a difference solely in how information on adverse effects is communicated could have a detectable effect on behavioral outcomes. A recent systematic review examined whether informing patients about benefits and harms of medicines compared to usual care has an impact on behavior at all [26]. Overall, the results did not show a significant effect. This systematic review had some limitations including heterogeneous results and statistical imprecision and there is some difficulty in interpreting the results. However, it suggests that we may need to focus on more general questions regarding the effects of provision of information on behavioral outcomes.

A further unanswered question is how different formats for describing the frequency of adverse effects are interpreted when they are presented together with treatment benefits, since these are also often overestimated by patients [27]. Qualitative research methods may be able to shed some light into how people come to assign probabilities to words. On a final note, further research should be conducted within the framework of a systematic review of the literature.

## Competing interests

All authors are purveyors and proponents of evidence based consumer health information. The authors did not receive any funding for this work apart from their salary.

## Authors’ contributions

RBB screened search results, extracted data, assessed studies for quality, performed the statistical analyses and drafted the manuscript. DF screened search results, extracted data and assessed study quality. AW extracted data, assessed study quality and contributed to statistical analyses. MK designed the search strategy and performed searches. ME screened search results. All authors participated in the design of the study and critically revised the manuscript. All authors read and approved the final manuscript.

## Pre-publication history

The pre-publication history for this paper can be accessed here:

http://www.biomedcentral.com/1472-6947/14/76/prepub

## Supplementary Material

Additional file 1 MEDLINE search strategy. Click here for file

Additional file 2 **Characteristics of included studies.** RCT = randomised controlled trial. Click here for file

Additional file 3 **Probability estimates for different wordings.** N/A = data not available. Click here for file

Additional file 4 PRIMSA Checklist. Click here for file
