# Supplementary material for: Words or numbers? Communicating risk of adverse effects in written consumer health information: a systematic review and meta-analysis
Source: BMC Med Inform Decis Mak. 2014 Aug 26;14:76. doi: 10.1186/1472-6947-14-76 (PMC4153005; doi:10.1186/1472-6947-14-76)
Supplement: Additional file 1 — MEDLINE search strategy. [file 1472-6947-14-76-S1.doc]

**Additional file 1: MEDLINE search strategy**

| #1 | randomized controlled trial.pt. |
| --- | --- |
| #2 | controlled clinical trial.pt. |
| #3 | comparative study.pt. |
| #4 | evaluation studies.pt. |
| #5 | clinical trial.pt. |
| #6 | cross?section$.mp. |
| #7 | survey.ti,ab. |
| #8 | (random$ adj allocat$).mp. |
| #9 | cross-sectional studies/ |
| #10 | randomized.ab. |
| #11 | placebo.ab. |
| #12 | clinical trials as topic.sh. |
| #13 | randomly.ab. |
| #14 | trial.ti. |
| #15 | ((random$ or control$) adj5 (trial$ or stud$)).ti,ab. |
| #16 | (cross$ adj section$ adj3 (trial$ or stud$)).ti,ab. |
| #17 | 1 or 2 or 3 or 4 or 5 or 6 or 8 or 9 or 10 or 11 or 12 or 13 or 14 or 15 or 16 |
| #18 | exp animals/ not humans.sh. |
| #19 | 17 not 18 |
| #20 | ((patient$1 or consumer$1 or written or print$ or health) adj2 (information or instruction$ or advice or advice$ or educat$)).ab,ti. |
| #21 | exp Drug Labeling/ |
| #22 | exp Drug Packaging/ |
| #23 | exp Pamphlets/ |
| #24 | exp Product Labeling/ |
| #25 | exp Patient Education as Topic/ |
| #26 | exp health education/ or consumer health information/ |
| #27 | 20 or 21 or 22 or 23 or 24 or 25 or 26 |
| #28 | ((wording or verbal$ or descript$ or communicat$ or present$) adj5 (benefit$ or harm$ or risk$ or (adverse adj1 (effect or effects or event or events)) or ((side or treatment) adj1 (effect or effects)))).ab,ti. |
| #29 | 19 and 27 and 28 |
| #30 | limit 29 to (english or german) |
